# Association of MDM2 expression with shorter progression-free survival and overall survival in patients with advanced pancreatic cancer treated with gemcitabine-based chemotherapy

**DOI:** 10.1371/journal.pone.0180628

**Published:** 2017-07-05

**Authors:** Shih-Hung Yang, Jen-Chieh Lee, Jhe-Cyuan Guo, Sung-Hsin Kuo, Yu-Wen Tien, Ting-Chun Kuo, Ann-Lii Cheng, Kun-Huei Yeh

**Affiliations:** 1 Departments of Oncology, National Taiwan University Hospital, Taipei, Taiwan; 2 Department of Internal Medicine, National Taiwan University Hospital, Taipei, Taiwan; 3 Graduate Institute of Clinical Medicine, National Taiwan University College of Medicine, Taipei, Taiwan; 4 Department of Pathology, National Taiwan University Hospital, Taipei, Taiwan; 5 Graduate Institute of Oncology, National Taiwan University College of Medicine, Taipei, Taiwan; 6 Department of Surgery, National Taiwan University Hospital, Taipei, Taiwan; 7 Department of Traumatology, National Taiwan University Hospital, Taipei, Taiwan; Universidade do Algarve Departamento de Ciencias Biomedicas e Medicina, PORTUGAL

## Abstract

This study evaluated the prognostic roles of murine double minute 2 (MDM2) and p53 in pancreatic cancer patients treated with gemcitabine-based chemotherapy. A total of 137 advanced or recurrent adenocarcinoma patients who were treated with gemcitabine-based palliative chemotherapy were reviewed, selected from 957 patients with pancreatic malignancy between 2008 and 2013 at our hospital. Immunohistochemical staining for MDM2 and p53 with formalin-fixed, paraffin-embedded tumor tissues was independently reviewed. Nuclear or cytoplasmic expression of MDM2 and p53 was found in tumor cells of 30 (21.9%) and 71 (51.8%) patients, respectively. Patients with MDM2 expression had shorter median overall survival (OS) (3.7 vs 5.8 mo; *P* = .048) and median progression-free survival (PFS) (1.5 vs 2.5 mo; *P* < .001); by contrast, p53 expression was not correlated with OS or PFS. In the multivariate analysis, MDM2 expression (hazard ratio = 1.731; *P* = .025) was an independent and unfavorable prognostic factor of OS. Additionally, MDM2 expression was significantly associated with progressive disease (PD) and death (*P* = .015) following first-line gemcitabine-based therapy. In advanced pancreatic cancer patients, MDM2 expression is associated with shorter OS and PFS after gemcitabine-based chemotherapy.

## Introduction

Pancreatic cancer is one of the leading causes of cancer-related mortalities in the world, resulting in more than 330000 deaths per year [1]. The 5-year overall survival (OS) rate is only 20% among patients receiving curative surgery and adjuvant gemcitabine, and patients with advanced diseases face even lower (< 5%) OS [2, 3]. Gemcitabine has been the most crucial element in the development of first-line chemotherapy since 1997 [3–6]. Following FOLFIRINOX establishing the role in first-line therapy for advanced pancreatic cancer [7], gemcitabine plus nab-paclitaxel also has become a new treatment standard for patients with favorable performance status (PS) [8]. Regarding the mechanisms of gemcitabine activation and metabolism, human equilibrative nucleoside transporter 1 represents the most consistent predictive biomarker for the efficacy of gemcitabine; however, data on other markers, such as deoxycytidine kinase and ribonucleotide reductase subunits 1 and 2, are heterogeneous [9]. The complex genetic background may largely contribute to the biology of pancreatic cancer and limit the utility of any single biomarker for drugs [10].

Gemcitabine, a nucleoside analogue, incorporates with DNA after activation, subsequently terminating DNA elongation [11]. After gemcitabine-induced DNA damage, p53 is activated and may contribute to apoptosis or cell cycle arrest [12, 13]. The chemosensitivity of gemcitabine in pancreatic cancer is enhanced after the restoration of p53 function [14]. However, p53 is mutated in more than 50% of pancreatic cancer cases [15], and MDM2, the negative regulator of p53, is induced and overexpressed by Ras signaling in pancreatic cancer [16]. MDM2 suppresses the transcriptional activity of p53 by binding to the transactivation domain of p53 [17]. In addition, MDM2 is an E3 ubiquitin ligase for p53 to mediate its degradation [18]. Therefore, functional p53-mediated apoptosis and cell cycle regulation may be inefficient, thus contributing little to gemcitabine-mediated cytotoxicity in pancreatic cancer patients. Furthermore, the status of p53 is not prognostic for pancreatic cancer [19–22], and the prognostic significance of MDM2 in resected pancreatic cancer is inconsistent [21, 22].

MDM2 exerts numerous other biological effects unrelated to p53, such as the regulation of p21, E2F1, XIAP, p73, and NF-κB/p65 [23–27]. In addition, the association between chemotherapy and MDM2 status in pancreatic cancer is largely unknown. In this study, we evaluated the prognostic values of MDM2 and p53 expression in advanced pancreatic cancer patients receiving gemcitabine-based palliative chemotherapy.

## Methods and materials

The cancer registry database of the Medical Information Management Office at National Taiwan University Hospital was screened for primary pancreatic malignancy diagnoses between 2008 and 2013. The patients selected for this study were required to have received palliative treatment with gemcitabine-containing chemotherapy (S1 Table) for advanced or recurrent pancreatic cancer; complete available medical records and histopathological archival tissues were also obtained. Patients with benign tumors, neuroendocrine tumors, solid pseudopapillary neoplasm, or pancreatic malignancies other than adenocarcinoma were excluded. In total, 137 patients who met our inclusion criteria were selected for analysis (S1 Fig). This study was approved by the Research Ethics Committee of National Taiwan University Hospital (approval number: 201309033RINB). Written consents were waived by the Research Ethics Committee. The dataset generated and/or analyzed during the current study was de-identified and available in the supplement.

### Immunohistochemistry

We applied immunohistochemical (IHC) staining to formalin-fixed, paraffin-embedded tumor tissue sections (4-μm thick), using the OptiView DAB IHC Detection Kit (Roche) and Ventana automated slide strainers (Roche). The primary antibodies and their dilutions comprised anti-MDM2 diluted to 1:100 (#33–7100, Invitrogen Corporation) and anti-p53 diluted to 1:50 (M 7001, Dako). Stained tissue sections were reviewed and scored by a pathologist (Jen-Chieh Lee) who is an expert in the interpretation of MDM2 expression [28] and was blinded to the patients’ demographic data and clinical outcomes. Expression was defined as positive when at least 10% of the tumor cells had positive staining [21]. The positive controls of p53 and MDM2 staining were colon adenoma and liposarcoma, respectively.

### Statistical analysis

Most (n = 130) selected patients were dead before initiation of this study, and the other seven patients without the confirmation of death also had been selected. OS was the primary endpoint in this study and was defined from the first day of gemcitabine-based chemotherapy to the day of death or final follow-up. Tumor response was evaluated according to the Response Evaluation Criteria in Solid Tumors (RECIST), Version 1.1 [29]. PFS was defined as imaging-documented PD with RECIST or death after initiation of a gemcitabine-based chemotherapy; therefore, progression was defined as PD or death after a gemcitabine-based chemotherapy.

A Fisher’s exact test was used to analyze the correlations between the discrete clinicopathologic characteristics and the IHC expression of MDM2 and p53. The association between MDM2 and p53 IHC expression was analyzed using the Fisher’s exact test. The prognostic significance of OS among the clinicopathologic factors and the expression of MDM2 and p53 were evaluated using univariate analysis and Kaplan—Meier survival curves (i.e., log-rank test). The clinicopathologic factors with significance in the univariate analysis were subsequently introduced into a multivariate analysis (i.e., Cox regression model) for OS. The cutoff point of OS data follow-up was July 2015.

The SPSS statistical software system (IBM SPSS Statistics for Windows, Version 20.0; IBM Corp., Armonk, NY, USA) was employed for statistical analyses, and *P* < .05 was considered statistically significant.

## Results

### Patient characteristics

Our analysis included 137 patients. The median age was 62 years (range: 27–84 y), and male patients comprised 60.6% (*n* = 83) of the study population. Most of the patients had favorable PS according to the criteria of the Eastern Cooperative Oncology Group (ECOG) 0–1 (81.0%, *n* = 111), and most of them were at the advanced stages of disease (stages III or IV, according to the American Joint Committee on Cancer) (74.5%, *n* = 102). Initially, of the 86 patients with stage IV disease, the most common metastatic sites were the liver (*n* = 69), peritoneum or omentum (*n* = 30), and lungs (*n* = 17). Patients in the first-line gemcitabine subgroup had comparable clinical characteristics comparing to the whole study cohort. The baseline patient characteristics are summarized in Table 1.

**Table 1:** Patient characteristics.

Characteristics	All patients	1^st^–line gemcitabine	Non-1^st^-line gemcitabine
	n = 137 (%)	n = 121 (%)	n = 16 (%)
Age			
median	62	62	63
range	27–84	27–84	46–73
Sex			
male	83 (60.6)	74 (61.2)	9 (56.3)
female	54 (39.4)	47 (38.8)	7 (43.8)
ECOG PS			
0–1	111 (81.0)	98 (81.0)	13 (81.3)
2–3	26 (19.0)	23 (19.0)	3 (18.8)
Stage*			
I	2 (1.5)	1 (0.8)	1 (6.3)
II	33 (24.1)	23 (19.0)	10 (62.5)
III	16 (11.7)	14 (11.6)	2 (12.5)
IV	86 (62.8)	83 (68.6)	3 (18.8)
T			
1–2	21 (15.3)	18 (14.9)	3 (18.8)
3	65 (47.4)	56 (46.3)	9 (56.3)
4	51 (37.2)	47 (38.8)	4 (25.0)
N			
0	60 (43.8)	56 (46.3)	4 (25.0)
1	77 (56.2)	65 (53.7)	12 (75.0)
Diabetes			
Yes	57 (41.6)	54 (44.6)	3 (18.8)
No	80 (58.4)	67 (55.4)	13 (81.3)
Cigarette smoking			
Yes	44 (32.1)	37 (30.6)	7 (43.8)
No	93 (67.9)	84 (69.4)	9 (56.3)
Primary			
head	66 (48.2)	55 (45.5)	11 (68.8)
neck or body	40 (29.2)	36 (29.8)	4 (25.0)
tail	31 (22.6)	30 (24.8)	1 (6.3)
Surgery			
none	79 (57.7)	76 (62.8)	3 (18.8)
curative	26 (19.0)	18 (14.9)	8 (50.0)
bypass	28 (20.4)	23 (19.0)	5 (31.3)
other	4 (2.9)	4 (3.3)	0
Radiotherapy			
Yes	20 (14.6)	13 (10.7)	7 (43.8)
No	117 (85.4)	108 (89.3)	9 (56.3)
Differentiation			
poor	44 (32.1)	42 (34.7)	2 (12.5)
moderate	72 (52.6)	62 (51.2)	10 (62.5)
good	21 (15.3)	17 (14.0)	4 (25.0)
CA 19–9 (U/mL)			
<500	59 (43.1)	54 (44.6)	5 (31.3)
≥500	70 (51.1)	60 (49.6)	10 (62.5)
unknown	8 (5.8)	7 (5.8)	1 (6.3)
CEA (ng/mL)			
<3	49 (35.8)	42 (34.7)	7 (43.8)
≥3	74 (54.0)	68 (56.2)	6 (37.5)
unknown	14 (10.2)	11 (9.1)	3 (18.8)
Hematology and biochemistry^¶^^#^			
WBC (per mm^3^)			
median	7,550	7,580	6,825
range	3,570–14,680	3,570–14,680	5,160–11,970
PMN (per mm^3^)			
median	5,352	5,554	4,187
range	1,911–13,315	1,911–13,315	2,673–10,390
Mono (per mm3)			
median	393	393	370
range	45–1,757	45–1,757	193–622
Lym (per mm^3^)			
median	1,409	1,398	1,640
range	338–3,691	338–3,691	498–3,580
Platelet (x10^3^; per mm^3^)			
median	237	237	241
range	67–539	67–539	136–410
CRP (mg/dL)			
median	1.93	1.93	2.48
range	0.07–21.21	0.07–21.21	0.56–4.40
Albumin (g/dL)			
median	4.3	4.3	4.3
range	0.8–5.3	2.3–5.3	0.8–4.9

*Stage: TNM system of the American Joint Committee on Cancer (7^th^ edition)

^¶^Hematology and biochemistry: WBC, white blood cell; PMN, polymorphonuclear granulocyte; Mono, monocyte; Lym, lymphocyte; CRP, C-reactive protein

^#^Missing data (patient number) in the whole study group: PMN, Mono, Lym (n = 8); CRP (n = 107); Albumin (n = 16)

In total, 26 patients received curative operations, 5 of whom were given adjuvant therapy with 5-FU-based chemotherapy and/or concurrent chemoradiotherapy; all patients experienced recurrence. As for palliative chemotherapy, gemcitabine had been used in the first-, second-, third-, or later-line therapy in 121, 39, 6, and 6 patients, respectively.

### IHC expression of MDM2 and p53 versus clinical characteristics

**Fig 1:**
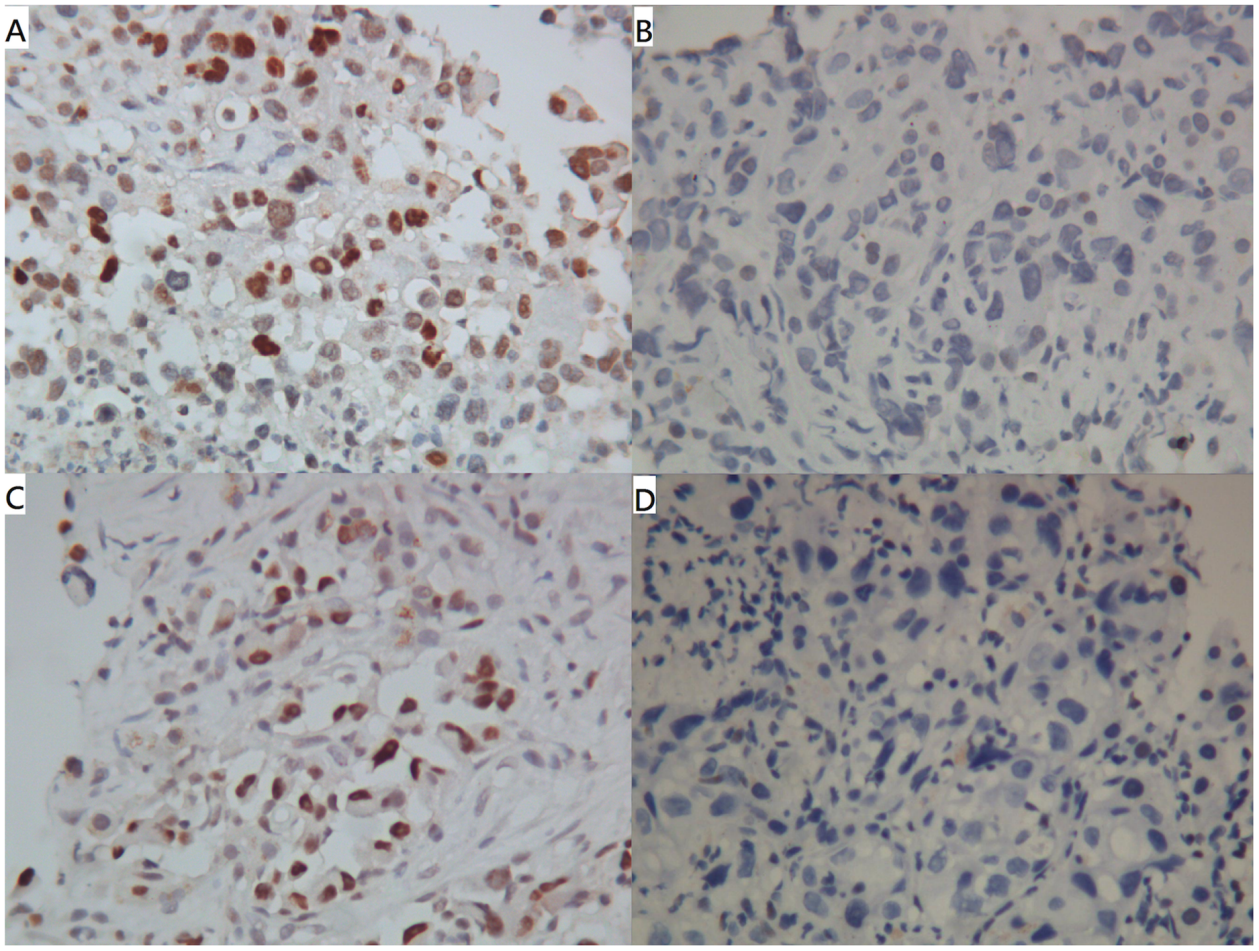
Representative cases of IHC expression (magnification 400X). Cases of IHC expression with (A) MDM2+, (B) MDM2-, (C) p53+, and (D) p53- were demonstrated. The positive staining was predominantly nuclear for both MDM2 and p53.

Nuclear or cytoplasmic expression of MDM2 and p53 was found in tumor cells of 30 (21.9%) and 71 (51.8%) cases, respectively (Fig 1A, 1B, 1C and 1D). The associations of MDM2 and p53 expression with patient clinical characteristics are presented in S2 Table. MDM2 or p53 expression was not significantly associated with any clinical factors. The association between the expression of MDM2 and p53 was not significant (*P* = .215). The positive rates of MDM2 and p53 expression stratified by the status of curative surgery did not show significant difference (S3 Table).

### Prognostic analyses

**Fig 2:**
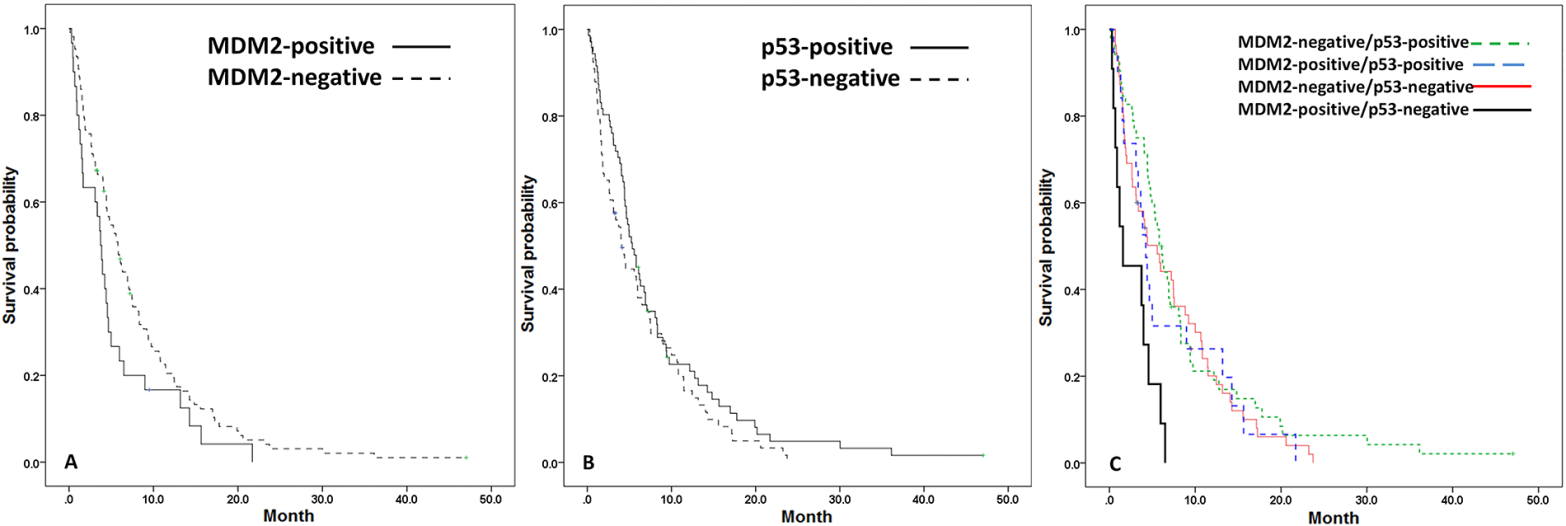
Survival curves in patients stratified with MDM2 and p53. The OS curves stratified with (A) MDM2 expression, (B) p53 expression, and (C) MDM2 and p53 statuses were demonstrated. The dots represented censored observation. The OS was worse in patients with MDM2+ IHC staining (*P* = .048). The OS did not differ significantly between p53+ and p53- patients (*P* = .192). After stratification of MDM2 and p53 status, patients with MDM2+/p53- staining had the shortest OS (*P* = .003).

For the entire study group, patients with MDM2 expression had significantly poorer prognosis than those without MDM2 expression did as calculated from the start of a gemcitabine-based regimen (median OS = 3.7 vs 5.8 mo; *P* = .048) (Fig 2A). By contrast, p53 expression had no prognostic significance (median OS = 5.3 vs 4.1 mo; *P* = .192) (Fig 2B). After stratification of all the patients into four subgroups (MDM2+/p53-, MDM2+/p53+, MDM2-/p53+, and MDM2-/p53-), the median OS following the start of a gemcitabine-based regimen was 1.6, 4.2, 5.8, and 5.6 months, respectively (*P* = .003; Fig 2C); within the same subgroups, patients with unresectable diseases (*n* = 111) demonstrated a median OS of 0.9, 4.2, 5.8, and 7.4 months (*P* = .001), respectively, following the start of a gemcitabine-based regimen. The association between MDM2 and poor OS was similar irrespective of surgery status (S4 Table).

In patients with stage III or IV pancreatic cancer and receiving first-line gemcitabine monotherapy (*n* = 36), MDM2 but not p53 was a poor prognostic factor (S5 Table).

In addition to MDM2 expression, age (*P* = .032), ECOG PS (*P* < .001), initial carcinoembryonic antigen (CEA) level (*P* = .024), and initial albumin level (*P* = .038) were all significantly associated with median OS from the start of any gemcitabine-based regimen in the univariate analyses (Table 2). Notably, the poor prognostic factors in the univariate analysis were not associated with any MDM2 or p53 subgroup. Moreover, after the significant clinical characteristics were introduced into the multivariate analysis (Table 3), only ECOG PS (HR = 5.032; *P* < .001) and expression of MDM2 (HR = 1.731; *P* = .025) remained unfavorable prognostic factors for OS from the start of a gemcitabine-based regimen.

**Table 2:** Univariate analysis for OS.

Characteristic	Value	Events	Median OS	SE*	P
Age (years)	≥60	74	4.4	0.5	**0.032**
	<60	56	5.6	1.1	
Sex	Male	77	5.0	0.9	0.395
	Female	53	4.6	1.0	
ECOG PS	0–1	104	6.0	0.8	<**0.001**
	2–3	26	1.6	0.1	
Stage	I/II/III	47	5.8	0.6	0.191
	IV	83	4.4	0.5	
T	1–3	80	4.6	0.6	0.540
	4	50	6.0	1.5	
N	0	56	4.6	0.9	0.751
	1	74	5.2	0.7	
Diabetes	No	75	5.0	0.7	0.471
	Yes	55	4.5	0.8	
Smoking	No	89	5.6	0.6	0.756
	Yes	41	4.2	0.3	
Primary	Tail	28	4.6	1.3	0.190
	Others	102	5.0	0.5	
Differentiation	Poor	43	4.0	0.7	0.057
	Good/Moderate	87	5.8	0.5	
CA 19–9 (U/mL)	≥500	65	4.4	0.3	0.117
	<500	57	5.8	0.5	
CEA (ng/mL)	≥3	71	4.6	0.3	**0.024**
	<3	46	5.6	1.5	
CRP (mg/dL)	≥1.5	18	3.6	0.8	0.057
	<1.5	11	5.8	4.0	
Albumin (g/dL)	≥4	83	6.0	0.8	**0.038**
	<4	31	3.4	0.6	
MDM2	positive	29	3.7	0.4	**0.048**
	negative	101	5.8	0.6	
p53	positive	67	5.3	0.7	0.192
	negative	63	4.1	0.6	
MDM2/p53	+/+ (N = 19)	18	4.2	0.5	**0.003**
	+/- (N = 11)	11	1.6	1.6	
	-/+ (N = 52)	49	5.8	0.6	
	-/- (N = 55)	52	5.6	1.1	

*SE: standard error

**Table 3:** Multivariate analysis for OS.

	Status		HR* (95% CI)	P
Characteristic	Unfavourable	Favourable		
Age (years)	≥60	<60	1.493 (0.967–2.304)	0.070
ECOG PS	2–3	0–1	5.032 (2.687–9.421)	<**0.001**
CEA (ng/mL)	≥3	<3	1.455 (0.921–2.299)	0.108
Albumin (g/dL)	<4	≥4	0.989 (0.583–1.680)	0.968
MDM2	positive	negative	1.731 (1.070–2.798)	**0.025**

*HR: hazard ratio

### IHC expression of MDM2 and p53 versus chemotherapy outcomes

**Fig 3:**
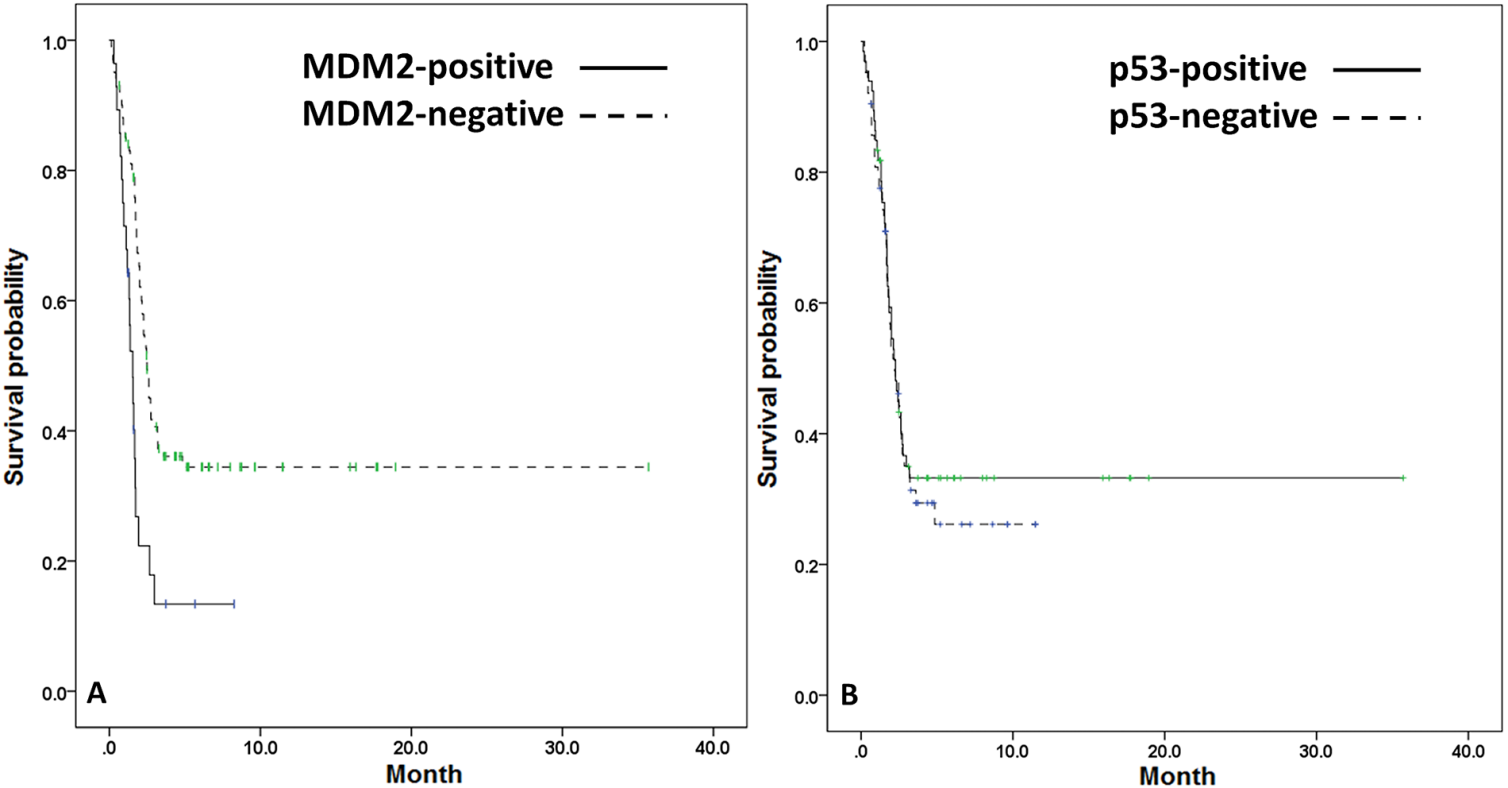
PFS curves in patients stratified with MDM2 and p53. The PFS curves were stratified with (A) MDM2 expression and (B) p53 expression. The median PFS was worse in patients with MDM2+ IHC staining (*P* < .001). The median PFS did not differ significantly between p53+ and p53- patients (*P* = .630). The dots represented censored observation.

We analyzed the association of best response to gemcitabine-based regimens and the expression of MDM2 and p53, but no significant association was observed in the entire study population (Table 4). The median PFS after initiation of any gemcitabine-based therapy in the entire study population was 2.3 months; furthermore, MDM2 expression was significantly associated with shorter median PFS (positive vs negative = 1.5 vs 2.5 mo; *P* < .001; Fig 3A) but p53 expression was not (positive vs negative = 2.3 vs 2.2 mo; *P* = .630; Fig 3B).

**Table 4:** MDM2 and p53 status versus chemotherapy response and outcome.

	MDM2		P	p53		P
	+	-		+	-	
Best response (*n* = 107*)			0.601			0.561
CR/PR/SD^¶^	6	38		25	19	
PD^¶^	12	51		32	31	
**Progression**						
Progression-free survival (month)						
All patients (*n* = 129*)	1.5	2.5	<**0.001**	2.3	2.2	0.630
1^st^ line (*n* = 114*)	1.4	2.5	<**0.001**	2.1	2.2	0.940
2^nd^ line (*n* = 37*)	1.7	3.2	0.279	2.2	3.6	0.619
1^st^ line (*n* = 114*)			**0.015**			1.000
No progression (CR/PR/SD) ^¶^	3	34		19	18	
Progression (PD/death) ^¶^	22	55		38	39	
2^nd^ line (*n* = 37*)			0.660			0.517
No progression (CR/PR/SD) ^¶^	2	16		8	10	
Progression (PD/death) ^¶^	4	15		11	8	

*evaluable patients

^¶^CR, complete response; PR, partial response; SD, stable disease; PD, progressive disease

We also stratified the patients according to their progression through gemcitabine-based therapy. MDM2 was significantly associated with progression after first-line gemcitabine-based therapy (*P* = .015) (Table 4).

## Discussion

In this study, MDM2 was determined to be a prognostic factor for poor prognosis and progression under gemcitabine-based chemotherapy in addition to other poor prognostic factors identified in a previous study, such as old age, poor ECOG PS, high CEA level, and low albumin level [30]. Although it was not clearly linked to any baseline characteristics associated with poor prognosis, MDM2 had borderline significance associated with negative regional lymph node involvement, which is generally a favorable prognostic factor. However, the lymph node status was not indicative of prognostic significance, regardless of curative resection. Previous studies have suggested that E-cadherin is a target for MDM2-mediated ubiquitination and degradation in breast cancer cells [31]; additionally, overexpression of MDM2 can inhibit cell—cell contact and increase cell motility [31]. Thus, although MDM2 may mediate distant nonregional lymph node metastasis through the downregulation of E-cadherin, the poor efficacy of systemic chemotherapy in the palliative setting actually outweighs the significance of regional lymph node metastasis.

The percentages of MDM2 and p53 IHC expression in this study were similar to those from previous reports [19, 21, 22]. Under ordinary conditions, MDM2 and p53 form a negative regulation loop [23]. MDM2 expression has been activated through the Ras—Raf—MEK pathway [16, 32], but the inverse relationship between MDM2 and p53 levels has not been observed in our study and in pancreatic cancer cell lines with mutant p53 [16]. Although MDM2 regulates the stability of mutant p53 in transgenic animal models [33], it also ubiquitinates mutant p53 less efficiently [34]. Therefore, both active Ras signaling and p53 mutation may partially contribute to the protection and decoupling of mutant p53 from MDM2-mediated degradation.

Furthermore, we determined that MDM2 expression, but not p53 expression, was associated with disease progression, poor PFS, and poor OS after gemcitabine-based chemotherapy. Notably, the shortest OS was observed in patients with MDM2+/p53- expression. Previous studies have also indicated that mutant p53 is associated with short OS, irrespective of curative resection [35, 36]. Recently, Fiorini *et al* observed that CDK1 and CCNB1 were induced after gemcitabine treatment in PANC1 cells expressing mutant p53 protein; however, they also noted that the effects were reversed after the downregulation of mutant p53 [37]. Conversely, in AsPC1 cells without expression of mutant p53 protein, the induction of CDK1 and CCNB1 expression occurred after the transfection of the mutant p53 (i.e., R273H) plasmid [37]. Therefore, the function gain that accompanies p53 mutation not only reverses cell cycle inhibition of wild-type p53 but also induces chemoresistance to gemcitabine in pancreatic adenocarcinoma cells.

In addition, p53 expression is not correlated with mutational status; this is also true of PANC1 and AsPC1 cells [37]. Because we did not incorporate p53 mutation analysis into the present study, we could not deduce the p53 mutation status of individual patients from the p53 IHC expression data. The four subgroups stratified by MDM2 and p53 IHC expression were not associated with poor prognostic factors. Notably, the two subgroups with extreme OS difference had opposite status of MDM2 and p53 expression. Therefore, we can assume that the balance between MDM2 and p53 mediates the tumor aggressiveness; as prior research similarly revealed, the downregulation of MDM2 in SW1990HM pancreatic adenocarcinoma cells increases levels of E-cadherin and decreases levels of matrix metallopeptidase 9 and Ki-67 [38]. Downregulation and induction of the autoubiquitination of MDM2 with SP141 inhibit pancreatic adenocarcinoma both *in vitro* and *in vivo*. Additionally, the induction of apoptosis, p21, and Bax, accompanied by a reduction of cyclin E and Bcl-2, occurs after SP141 treatment [39]. Although MDM2 amplification is among the mechanisms of MDM2 expression in specific malignancies [40], a typical pattern of the phenomenon was not found in this study (S2 Fig). Therefore, MDM2 amplification was not the major mechanism of MDM2 expression in our patient population, which aligns with previous studies reporting rare MDM2 amplification in pancreatic adenocarcinoma [41].

There were some missing data in Table 1 due to the limitation of retrospective study. Most patients did not have baseline data of CRP. Although the level of CRP may be associated with prognosis, it was not a routine test at the diagnosis of pancreatic cancer. The distribution of baseline characteristics, MDM2 and p53 expression was similar among the entire study population and subjects without missing data of CEA, CA 19–9, polymorphonuclear granulocyte (PMN) count, monocyte count, and lymphocyte count (S6 Table). In addition, the multivariate analysis of the subjects without missing data (S7 Table) was comparable to the original analysis (Table 3).

## Conclusions

In summary, MDM2 expression is associated with poor prognosis and progression after gemcitabine-based chemotherapy in advanced pancreatic adenocarcinoma. The major limitation of our study was the heterogeneous patient population, comprising patients both with and without curative resection. However, all of these patients had been previously treated with gemcitabine. To the best of our knowledge, this is the first clinical study to evaluate the association of chemotherapy with MDM2 in pancreatic cancer. Future basic or clinical studies applying chemotherapy and MDM2-targeted therapy with a non-p53 dependent mechanism are warranted.

## Supporting information

S1 Fig Consort diagram of eligible patients. The process of patient selection was demonstrated.
(TIFF)

S2 Fig FISH patterns in patients with polysomy of chromosome 12. FISH patterns in the three patients with polysomy of chromosome 12 with concomitant increase numbers of the centromere and mdm2 staining [27] were demonstrated; red = MDM2; green = centromere 12.
(TIF)

S1 Table Summary of first-line and second-line gemcitabine-based chemotherapy. (DOC)

S2 Table Association of MDM2 and p53 with clinical characteristics. (DOC)

S3 Table Association of MDM2/p53 expression and surgery status. (DOC)

S4 Table Association of MDM2/p53 expression, surgery status, and OS after gemcitabine-based chemotherapy. (DOC)

S5 Table Association of MDM2/p53 and PFS/OS in stage III/IV patients with first-line gemcitabine (N = 36). (DOC)

S6 Table Clinical characteristics and missing data. (DOC)

S7 Table Multivariate analysis for OS in subjects without missing data. (DOC)

S1 File Supplement original dataset. (XLS)
