# Supplementary figures and images for: Association of MDM2 expression with shorter progression-free survival and overall survival in patients with advanced pancreatic cancer treated with gemcitabine-based chemotherapy
Source: PLoS One. 2017 Jul 5;12(7):e0180628. doi: 10.1371/journal.pone.0180628 (PMC5498069; doi:10.1371/journal.pone.0180628)

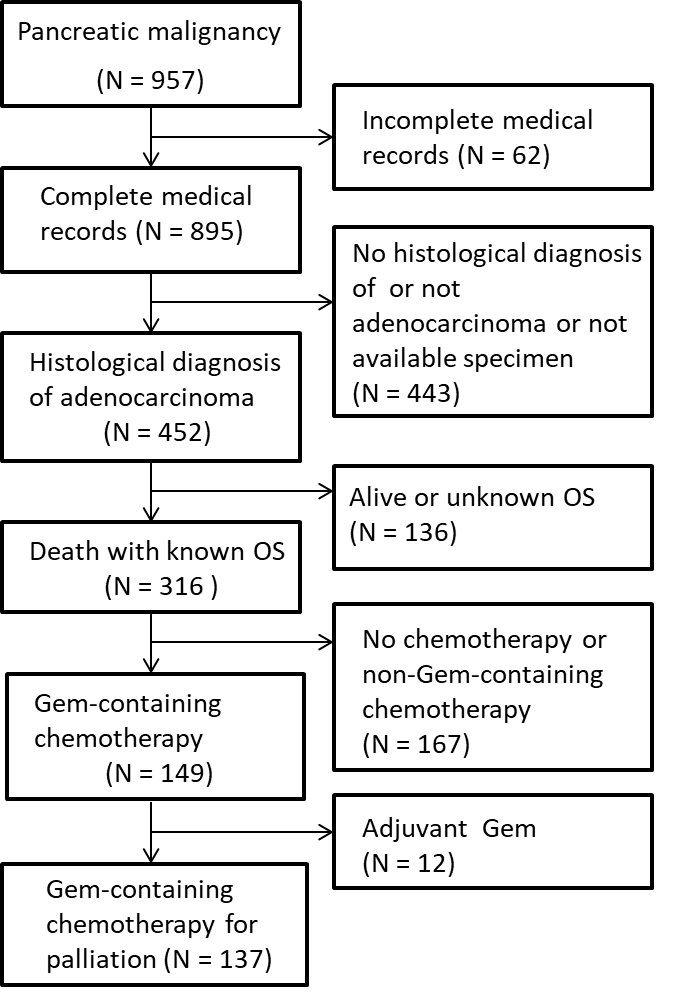

Supplement: S1 Fig — The process of patient selection was demonstrated. (TIFF) [file pone.0180628.s001.tiff]

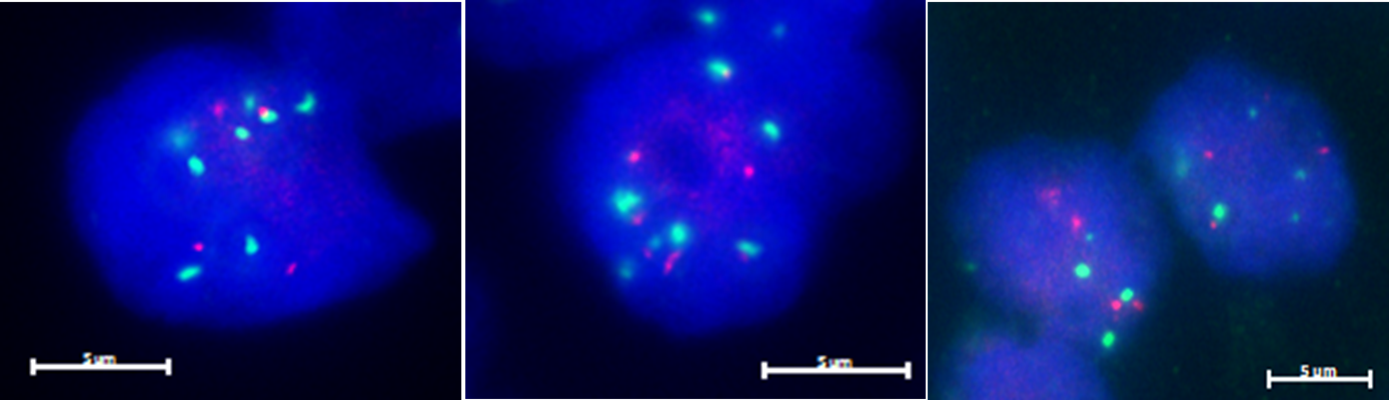

Supplement: S2 Fig — FISH patterns in the three patients with polysomy of chromosome 12 with concomitant increase numbers of the centromere and mdm2 staining [27] were demonstrated; red = MDM2; green = centromere 12. (TIF) [file pone.0180628.s002.tif]
