# Supplementary material for: Association of MDM2 expression with shorter progression-free survival and overall survival in patients with advanced pancreatic cancer treated with gemcitabine-based chemotherapy
Source: PLoS One. 2017 Jul 5;12(7):e0180628. doi: 10.1371/journal.pone.0180628 (PMC5498069; doi:10.1371/journal.pone.0180628)
Supplement: S1 Table — (DOC) [file pone.0180628.s003.doc]

**S1 Table. Summary of first-line and second-line gemcitabine-based chemotherapy**

| **Regimen** | **N** | |
| --- | --- | --- |
|  | **First-line** | **Second-line** |
| G alone | 44 | 7 |
| G/5-FU analog^*^ | 36 | 21 |
| G/Platinum^¶^ | 10 | 3 |
| G/Platinum^¶^/5-FU analog^*^ | 15 | 4 |
| G/Erlotinib | 13 | 4 |
| G/Others | 3 | 0 |
| ^*^5-FU analog: 5-FU, tegafur/uracil, capecitabine, or S-1 | | |
| ^¶^Platinum: cisplatin or oxaliplatin | | |
