# Supplementary material for: Association of MDM2 expression with shorter progression-free survival and overall survival in patients with advanced pancreatic cancer treated with gemcitabine-based chemotherapy
Source: PLoS One. 2017 Jul 5;12(7):e0180628. doi: 10.1371/journal.pone.0180628 (PMC5498069; doi:10.1371/journal.pone.0180628)
Supplement: S2 Table — (DOC) [file pone.0180628.s004.doc]

**S2 Table. Association of MDM2 and p53 with clinical characteristics**

| **Characteristic** | **Value** | **MDM2** | | **P** | **p53** | | **P** |
| --- | --- | --- | --- | --- | --- | --- | --- |
|  |  | **+** | **-** |  | **+** | **-** |  |
| Age (years) | ≥60 | 17 | 61 | 1.000 | 38 | 40 | 0.490 |
|  | <60 | 13 | 46 |  | 33 | 26 |  |
| Sex | Male | 20 | 63 | 0.528 | 42 | 41 | 0.730 |
|  | Female | 10 | 44 |  | 29 | 25 |  |
| ECOG PS | 0-1 | 24 | 87 | 1.000 | 61 | 50 | 0.190 |
|  | 2-3 | 6 | 20 |  | 10 | 16 |  |
| Stage | I/II/III | 8 | 43 | 0.205 | 28 | 23 | 0.600 |
|  | IV | 22 | 64 |  | 43 | 43 |  |
| T | 1-3 | 21 | 65 | 0.399 | 46 | 40 | 0.724 |
|  | 4 | 9 | 42 |  | 25 | 26 |  |
| N | 0 | 18 | 42 | 0.060 | 27 | 33 | 0.172 |
|  | 1 | 12 | 65 |  | 44 | 33 |  |
| Diabetes | No | 18 | 62 | 1.000 | 40 | 40 | 0.729 |
|  | Yes | 12 | 45 |  | 31 | 26 |  |
| Smoking | No | 17 | 76 | 0.184 | 46 | 47 | 0.467 |
|  | Yes | 13 | 31 |  | 25 | 19 |  |
| Primary | Tail | 7 | 24 | 1.000 | 15 | 16 | 0.688 |
|  | Others | 23 | 83 |  | 56 | 50 |  |
| Differentiation | Poor | 10 | 34 | 1.000 | 25 | 19 | 0.467 |
|  | Good/Moderate | 20 | 73 |  | 46 | 47 |  |
| CA 19-9 (U/mL) | ≥500 | 17 | 53 | 0.674 | 35 | 35 | 0.596 |
|  | <500 | 12 | 47 |  | 33 | 26 |  |
| CEA (ng/mL) | ≥3 | 15 | 59 | 0.511 | 37 | 37 | 0.587 |
|  | <3 | 13 | 36 |  | 27 | 22 |  |
| Liver mets | Yes | 20 | 49 | 0.217 | 37 | 32 | 0.279 |
|  | No | 2 | 15 |  | 6 | 11 |  |
| Peritoneum or omentum mets | Yes | 7 | 23 | 0.800 | 16 | 14 | 0.821 |
|  | No | 15 | 41 |  | 27 | 29 |  |
| Lung mets | Yes | 4 | 13 | 1.000 | 7 | 10 | 0.589 |
|  | No | 18 | 51 |  | 36 | 33 |  |
| p53 | Positive | 19 | 52 | 0.215 |  | | |
|  | Negative | 11 | 55 |  |  |  |  |
