# Supplementary material for: Association of MDM2 expression with shorter progression-free survival and overall survival in patients with advanced pancreatic cancer treated with gemcitabine-based chemotherapy
Source: PLoS One. 2017 Jul 5;12(7):e0180628. doi: 10.1371/journal.pone.0180628 (PMC5498069; doi:10.1371/journal.pone.0180628)
Supplement: S3 Table — (DOC) [file pone.0180628.s005.doc]

**S3 Table. Association of MDM2/p53 expression and surgery status**

| Marker | | MDM2 | | p53 | |
| --- | --- | --- | --- | --- | --- |
| Expression | | Positive | Negative | Positive | Negative |
| Curative surgery | | N (%) | | N (%) | |
|  | Yes | 4 (15.4) | 22 (84.6) | 12 (46.2) | 14 (53.8) |
|  | No | 26 (23.4) | 85 (76.6) | 59 (53.2) | 52 (46.8) |
| P | | 0.441 | | 0.663 | |
