# Supplementary material for: Association of MDM2 expression with shorter progression-free survival and overall survival in patients with advanced pancreatic cancer treated with gemcitabine-based chemotherapy
Source: PLoS One. 2017 Jul 5;12(7):e0180628. doi: 10.1371/journal.pone.0180628 (PMC5498069; doi:10.1371/journal.pone.0180628)
Supplement: S4 Table — (DOC) [file pone.0180628.s006.doc]

**S4 Table. Association of MDM2/p53 expression, surgery status, and OS after gemcitabine-based chemotherapy**

| Curative surgery | | Yes | | No | |
| --- | --- | --- | --- | --- | --- |
| N | | 26 | | 111 | |
|  | | Median OS* | P | Median OS* | P |
|  |  | (95% CI) |  | (95% CI) |  |
| MDM2 | |  | 0.253 |  | 0.143 |
|  | Positive | 1.6 |  | 3.7 |  |
|  |  | (0-4.5) |  | (2.6-4.8) |  |
|  | Negative | 4.1 |  | 6.1 |  |
|  |  | (2.0-6.2) |  | (4.7-7.5) |  |
| p53 | |  | 0.021 |  | 0.822 |
|  | Positive | 5.0 |  | 5.3 |  |
|  |  | (0-24.9) |  | (4.1-6.6) |  |
|  | Negative | 3.1 |  | 4.6 |  |
|  |  | (0-7.3) |  | (1.7-7.5) |  |
| *Median OS (months) | | | | | |
