# Supplementary material for: Association of MDM2 expression with shorter progression-free survival and overall survival in patients with advanced pancreatic cancer treated with gemcitabine-based chemotherapy
Source: PLoS One. 2017 Jul 5;12(7):e0180628. doi: 10.1371/journal.pone.0180628 (PMC5498069; doi:10.1371/journal.pone.0180628)
Supplement: S5 Table — (DOC) [file pone.0180628.s007.doc]

**S5 Table. Association of MDM2/p53 and PFS/OS in stage III/IV patients with first-line gemcitabine (N =36)**

|  | | N | Median OS* | P | Median PFS* | P |
| --- | --- | --- | --- | --- | --- | --- |
|  |  |  | (95% CI) |  | (95% CI) |  |
| MDM2 | |  |  | <0.001 |  | 0.028 |
|  | Positive | 10 | 0.9 |  | 0.7 |  |
|  |  |  | (0-1.9) |  | (0.5-0.9) |  |
|  | Negative | 26 | 5.2 |  | 1.9 |  |
|  |  |  | (3.2-7.3) |  | (1.4-2.4) |  |
| p53 | |  |  | 0.244 |  | 0.331 |
|  | Positive | 17 | 4.0 |  | 1.5 |  |
|  |  |  | (2.2-5.8) |  | (0.8-2.3) |  |
|  | Negative | 19 | 3.4 |  | 1.8 |  |
|  |  |  | (2.0-4.8) |  | (1.4-2.2) |  |
| *Median OS (months); PFS (month) | | | | | | |
