# Supplementary material for: Association of MDM2 expression with shorter progression-free survival and overall survival in patients with advanced pancreatic cancer treated with gemcitabine-based chemotherapy
Source: PLoS One. 2017 Jul 5;12(7):e0180628. doi: 10.1371/journal.pone.0180628 (PMC5498069; doi:10.1371/journal.pone.0180628)
Supplement: S6 Table — (DOC) [file pone.0180628.s008.doc]

**S6 Table. Clinical characteristics and missing data**

| **Characteristics** | | **All patients** | **Subjects without missing data** |
| --- | --- | --- | --- |
|  |  | **n = 137 (%)** | **n = 102 (%)** |
| Age | |  |  |
|  | median | 62 | 62 |
|  | range | 27-84 | 27-84 |
| Sex | |  |  |
|  | male | 83 (60.6) | 59 (57.8) |
|  | female | 54 (39.4) | 43 (42.2) |
| ECOG PS | |  |  |
|  | 0-1 | 111 (81.0) | 83 (81.4) |
|  | 2-3 | 26 (19.0) | 19 (18.6) |
| Stage* | |  |  |
|  | I | 2 (1.5) | 2 (2.0) |
|  | II | 33 (24.1) | 25 (24.5) |
|  | III | 16 (11.7) | 11 (10.8) |
|  | IV | 86 (62.8) | 64 (62.7) |
| T | |  |  |
|  | 1-2 | 21 (15.3) | 17 (16.7) |
|  | 3 | 65 (47.4) | 47 (46.1) |
|  | 4 | 51 (37.2) | 38 (37.3) |
| N | |  |  |
|  | 0 | 60 (43.8) | 43 (42.2) |
|  | 1 | 77 (56.2) | 59 (57.8) |
| Diabetes | |  |  |
|  | Yes | 57 (41.6) | 42 (41.2) |
|  | No | 80 (58.4) | 60 (58.8) |
| Cigarette smoking | |  |  |
|  | Yes | 44 (32.1) | 31 (30.4) |
|  | No | 93 (67.9) | 71 (69.6) |
| Primary | |  |  |
|  | head | 66 (48.2) | 48 (47.1) |
|  | neck or body | 40 (29.2) | 31 (30.4) |
|  | tail | 31 (22.6) | 23 (22.5) |
| Surgery | |  |  |
|  | none | 79 (57.7) | 56 (54.9) |
|  | curative | 26 (19.0) | 19 (18.6) |
|  | bypass | 28 (20.4) | 24 (23.5) |
|  | other | 4 (2.9) | 3 (2.9) |
| Radiotherapy | |  |  |
|  | Yes | 20 (14.6) | 15 (14.7) |
|  | No | 117 (85.4) | 87 (85.3) |
| Differentiation | |  |  |
|  | poor | 44 (32.1) | 32 (31.4) |
|  | moderate | 72 (52.6) | 51 (50.0) |
|  | good | 21 (15.3) | 19 (18.6) |
| CA 19-9 (U/mL) | |  |  |
|  | <500 | 59 (43.1) | 46 (45.1) |
|  | ≥500 | 70 (51.1) | 58 (54.9) |
|  | unknown | 8 (5.8) | 0 |
| CEA (ng/mL) | |  |  |
|  | <3 | 49 (35.8) | 41 (40.2) |
|  | ≥3 | 74 (54.0) | 61 (59.8) |
|  | unknown | 14 (10.2) | 0 |
| MDM2 | |  |  |
|  | Positive | 30 (21.9) | 23 (22.5) |
|  | Negative | 107 (78.1) | 79 (77.5) |
| P53 | |  |  |
|  | Positive | 71 (51.8) | 52 (51.0) |
|  | Negative | 66 (48.2) | 50 (49.0) |
| Hematology and biochemistry^¶＃^ | | | |
| WBC (per mm^3^) | |  |  |
|  | median | 7,550 | 7,460 |
|  | range | 3,570-14,680 | 3,570-14,680 |
| PMN (per mm^3^) | |  |  |
|  | median | 5,352 | 5,222 |
|  | range | 1,911-13,315 | 1,911-13,315 |
| Mono (per mm3) | |  |  |
|  | median | 393 | 377 |
|  | range | 45-1,757 | 45-1,757 |
| Lym (per mm^3^) | |  |  |
|  | median | 1,409 | 1,442 |
|  | range | 338-3,691 | 438-3,691 |
| Platelet (x10^3^; per mm^3^) | |  |  |
|  | median | 237 | 235 |
|  | range | 67-539 | 67-539 |
| CRP (mg/dL) | |  |  |
|  | median | 1.93 | 1.93 |
|  | range | 0.07-21.21 | 0.07-17.20 |
| Albumin (g/dL) | |  |  |
|  | median | 4.3 | 4.3 |
|  | range | 0.8-5.3 | 0.8-5.3 |
| *Stage: TNM system of the American Joint Committee on Cancer (7^th^ edition) | | | |
| ^¶^Hematology and biochemistry: WBC, white blood cell; PMN, polymorphonuclear granulocyte; Mono, monocyte; Lym, lymphocyte; CRP, C-reactive protein | | | |
