# Supplementary material for: Association of MDM2 expression with shorter progression-free survival and overall survival in patients with advanced pancreatic cancer treated with gemcitabine-based chemotherapy
Source: PLoS One. 2017 Jul 5;12(7):e0180628. doi: 10.1371/journal.pone.0180628 (PMC5498069; doi:10.1371/journal.pone.0180628)
Supplement: S7 Table — (DOC) [file pone.0180628.s009.doc]

**S7 Table. Multivariate analysis for OS in subjects without missing data**

|  | **Status** | | **HR*** |  |
| --- | --- | --- | --- | --- |
| **Characteristic** | **Unfavourable** | **Favourable** | **(95% CI)** | **P** |
| Age (years) | ≥60 | <60 | 1.409 | 0.131 |
|  |  |  | (0.903-2.199) |  |
| ECOG PS | 2-3 | 0-1 | 5.030 | **<0.001** |
|  |  |  | (2.666-9.492) |  |
| CEA (ng/mL) | ≥3 | <3 | 1.366 | 0.187 |
|  |  |  | (0.860-2.172) |  |
| Albumin (g/dL) | <4 | ≥4 | 1.070 | 0.808 |
|  |  |  | (0.619-1.849) |  |
| MDM2 | Positive | Negative | 1.735 | **0.028** |
|  |  |  | (1.060-2.839) |  |
| *HR: hazard ratio | | | | |
